# Female career interruption and social integration: An interaction between human capital and new media use

**DOI:** 10.3389/fpsyg.2022.917582

**Published:** 2022-08-24

**Authors:** Chunyan Li, Yongjin Liu, Weiming Li

**Affiliations:** School of Management, Hainan University, Haikou, China

**Keywords:** social integration, human capital, new media use, female career interruption, moderating analysis

## Abstract

Promoting the social integration of various groups provides a significant guarantee for China to achieve high-quality development. However, female workers, as the main force of the service industry, had suffered a greater occupational impact due to COVID-19 and loosened China’s fertility policy in 2021. After female career interruption, the change in women’s social roles and networks has aroused concern about their social integration. However, limited attention had been paid to female career interruption in existing studies about social integration. Therefore, this study developed a theoretical model to explore the relationship between female career interruption and social integration. An empirical test using data from the fourth Chinese Women’s Social Status Survey of Hainan Province was applied to evaluate the hypotheses. The results indicate that there is a significantly negative correlation between female career interruption and social integration. And there is a difference among female career interrupters with different quantiles of social integration. Furthermore, the impact of human capital on the link between female career interruption and social integration depended on the type of new media use. When female career interrupters who use strong learning-oriented new media (such as learning about news, working/business, studying online, and investing/financial management) encounter human capital, the relationship between female career interruption and social integration is minimal. In contrast, this relationship is enhanced when female career interrupters who use strong amusing-oriented new media (such as online consumption, chatting, entertainment, and games) are confronted with human capital. Meanwhile, the results of quantile regression show that the secondary moderating effect of learning-oriented new media use gradually weakens with the rise of the quantile of female social integration. And so does amusing-oriented new media use. However, a moderating effect of human capital alone is not found.

## Introduction

With the rapid development of China’s economy and the accelerating process of urbanization, promoting the social integration of various groups and creating a favorable population environment is not only an inherent requirement for achieving people’s yearning for a better life but also a significant guarantee to boost China’s high-quality development. At present, the adjustment and upgrading of China’s economic structure continue to accelerate. In 2020, the proportion of the service industry in China’s GDP had reached 54.5% (National Bureau of Statistics, 2021). There is a huge space for the development of Chinese female workers in the service industry, for which the employment proportion of women in the service industry is expected to exceed 60%. Moreover, this figure is expected to reach above 80% in 2050 (All China Women’s Federation, 2022). However, the career development of female workers in China has been still constrained by multiple factors. Specifically, due to China’s long-standing restrictions on fertility, China’s demographic dividend had disappeared and its population structure had been rapidly aging (Shen et al., 2012). To break this deadlock, China proposed the policy of “guiding families with only one child to have another child” in 2013, the “Two-child Policy” in 2015, and the “Three-child Policy” in 2021. Therefore, women are given higher fertility expectations by society because of the loosened fertility policy, which means that more women may have their careers interrupted in consideration of childbirth. Hofstede et al. (2005) rated more than 40 countries based on cultural dimension theory, and China was classified as a country with masculinity. In this context, influenced by traditional culture, Chinese women are often given the expectation of “good mothers” by the society to “rearing children and keeping husbands in clean shirts.” Cai et al. (2021) found in the follow-up survey of more than 5,600 practitioners during COVID-19 that women accounted for 78% of the unemployed in November 2020, 68% were married women, and 82% of the married group were unemployed women with at least one child. Accordingly, working mothers are facing a greater employment shock. Meanwhile, the pressure from gender segregation in the workplace makes women a vulnerable group in the workplace (Reskin, 1993; Charles and Grusky, 2005), who are more prone to career interruption. Overall, the problem of female career interruption is more severe than ever. A taste of an orderly career enhanced the vitality of participation (Wilensky, 1961). Once a woman’s career is interrupted, her social role changes from an employee to an unemployed, and her network and interpersonal objects also changed from the original work, life, and other multiple circumstances. Thus, the issue of women’s social integration has also become more prominent. However, departed studies on social integration mainly focused on migrants (Snel et al., 2006), migrant workers, and other floating populations (Yang, 2009). Less attention has been paid to female career interrupters. In addition, most existing studies on the factors affecting social integration were limited to human capital, social capital, and institutional structure (Li and Tian, 2012), while the research on the relationship between career interruption and social integration was very limited. Therefore, how to help female career interrupters reintegrate into society and participate in economic, social, and cultural life without exclusion has become the key to solving the social integration in this study.

The relationship between female career interruption and social integration can be influenced by various factors. Human capital is one of such factors. Human capital levels vary, as do human and social resources at one’s disposal (Qian et al., 2018), which directly contributes to the gap in their social integration abilities (Corak, 2013). Female workers with different levels of human capital experience dissimilar social integration upon returning to work after a career interruption. In addition, career skills, accumulated or lost during career interruption, can also have a powerful impact on social reintegration. As an essential means of learning and entertainment, new media plays an important role as a double-edged sword in the reintegration process, which cannot be ignored. People supplement their skills and knowledge promptly through online learning to meet the constant evolution of the external environment (Nygren et al., 2019), while some indulge in the virtual online world and gradually lose the ability to communicate with the outside world (Mortensen and Neeley, 2012). Therefore, it is necessary to further clarify the role of human capital and new media use when exploring the link between female career interruption and social integration.

Based on the foregoing analysis, we developed a theoretical model and use quantile regression to study the relationship between female career interruption and social integration. Moreover, we explore the effect of human capital on the relationship between female career interruption and social integration. Furthermore, we determine whether differently oriented new media use can cause differences in this process.

## Literature review and research hypotheses

### Female career interruption and social integration

Social integration was first defined as different ethnic groups sharing experiences and acquiring each others’ cognitive memory, emotional experience, and attitude, finally integrating into one cultural community (Park, 1928). Cameron (2006) defined social integration as a process of participating in social activities and being valued in the socio-economic, political, and psychological aspects while gaining respect and mutual trust. There is a lack of a unified definition of social integration in academics. In this study, social integration is interpreted as a dynamic process in which individuals get normal economic, political, public services, and other resources by integrating into mainstream social networks. Although there is no unified indicator for measuring social integration, scholars have reached a consensus to divide social integration into multiple dimensions (Liang, 2016). Representative dimensions include “two dimensions” (Gordon, 1964), “three dimensions” (Junger-Tas, 2001), and “four dimensions” (Entzinger, 2003). The social integration measures used by most Chinese scholars generally focus on economic, cultural, and psychological dimensions. Based on the perspective of social equity and social participation, social integration emphasizes social participation without exclusion, in addition to citizens’ fair enjoyment of rights (Burchardt et al., 2002), which represents the institutional requirements of social integration. Therefore, this study divided social integration into four dimensions: economic, cultural, psychological, and institutional.

A taste of an orderly career promoted social integration (Wilensky, 1961). However, there is limited research about the relationship between career interruption and social integration. Women are the focus of career interruption research (De Freitas et al., 2011). Arun et al. (2004) defined career interruption as a continuous interruption for women over 5 years. Drasch (2013) closely linked career interruption with family and defined career interruption as the phenomenon of mothers leaving work to take care of children after a baby’s birth. However, Ziefle and Gangl (2014) criticized that the lack of a clear definition of the time range of career interruption would affect the effectiveness of the research results. Huang (2014) defined career interruption as the experience of not working and having no labor income for more than half a year based on the Third Chinese Women’s Social Status Survey. This study aims to investigate female career interruption based on the Chinese cultural context. Therefore, referring to the definition of Huang (2014), female career interruption is defined as women voluntarily or forced to withdraw from the labor market without income or job for more than 6 months.

Career interruption involved the separation from the workplace and the change of communication groups, which conflicted with the ideal complete career development (Aisenbrey et al., 2009). Based on social integration theory, social integration focused on the relevance and population dynamics between individuals and the mainstream group (Friedkin, 2004), which can be affected by multiple factors such as occupational characteristics (Li and Tian, 2012) and group identity (Friedkin, 2004). Therefore, the occurrence of career interruption has a profound impact on the social integration of individuals. The following is a specific analysis of the four dimensions of social integration. For female workers, career interruption was a unique mother career trajectory embedded in the female life cycle that can indelibly mark a woman’s career (Cukrowska-Torzewska and Matysiak, 2020). Meanwhile, female career interruption increased the risk of female career downward mobility and reduced the opportunity for career upward mobility (Engelhardt et al., 2004; Mandel and Semyonov, 2006). The negative impact of female career interruption was directly reflected in “wage punishment” (Viitanen, 2014; Ali et al., 2020). Once a woman’s career was interrupted, her social role changed from an employee to an unemployed, and her relationship network and interpersonal objects also changed from the original work, life, and other multiple situations. Meanwhile, female career interrupters faced multiple pressures of family-work conflict (Hammer et al., 2003) and career development (Mandel and Semyonov, 2006), which consumed their existing energy and increase their psychological costs. In particular, female reproductive career interruption solidified the existing gender segregation in the workplace, and discrimination by employers accompanied the whole life throughout her career (Li, 2015). Especially in the context of COVID-19, the anxiety, depression, and stress of the Chinese increased significantly during the “lockdown” period (Wang et al., 2020). The COVID-19 epidemic further magnified the psychological problems of the majority of female career interrupters. Therefore, the link between female career interruption and economic and psychological integration will be negative.

Previous studies revealed that stable employment ensured that employees could have access to stable social networks (Xie, 2015). The longer the employees stayed on the job, the more opportunities they had to contact different groups in that daily communication helped to keep information flowing (Shi and Zhu, 2014). In addition, the labor law requires that organizations should purchase social security for on-the-job employees to provide institutional security for them. On the contrary, the career gap during female career interruption deprived them of the convenience of exchanging information with the external world. Due to information asymmetry, female social activities and social participation were both limited. Potentially, it reduced female career interrupters’ expectations and actual participation in culture, widening the gap with the mainstream cultural identity, and finally formed an invisible barrier to cultural integration. Meanwhile, those women who withdrew from the workplace due to career interruption no longer enjoyed the institutional guarantee provided by previous organizations. Thus, female career interrupters’ obligations, rights, and guarantees were hard to achieve full equivalence with the mainstream social group. Finally, this resulted in blocked institutional integration. In summary, female career interruption hindered economic, cultural, psychological, and institutional integration, which, in turn, affected social integration. Based on the aforementioned theoretical analysis, the first research hypothesis is proposed.

**Hypothesis 1:** The relationship between female career interruption and social integration is negative. The degree of female social integration will decrease with the occurrence of female career interruption.

### Moderating role of human capital

Organization for Economic Co-operation and Development (2001) defines human capital as the knowledge, skills, abilities, and qualities possessed by individuals that can create personal, social and economic well-being. Referring to the definition of OECD, our study defines female human capital as the sum of economically valuable knowledge, technology, ability, and fitness contained in women through investment.

Human capital is the reflection of an individual’s ability to integrate into society and affects the degree of social integration through integrating willingness (Wang et al., 2018). Huang et al. (2018) found that rural women could increase their income by more than 98% after higher education. Long (1980) and Borjas (1982) noticed that improving knowledge and skills helped to increase return on revenue and promote social integration. If investment in female human capital increases, income growth can effectively improve female economic integration. Women with strong human capital are usually well-educated, skilled, and physically healthy. Skill training is also an important way to enhance human capital. Such women have better access to high-quality employment after career interruption. This somewhat offsets the economic integration of career interruption. Meanwhile, when individuals’ education, health and other human capital improved, the subjective mindset was more conducive to survival (Zhu, 2019). In other words, they were more willing to accept new things (Dustmann, 1996). Therefore, strong human capital can enhance female cultural acceptance, improve their cultural adaptability, and weaken the negative link between female career interruption and cultural integration. From the perspective of social exclusion, improved education effectively reduced the possibility of unfair treatment (Liu et al., 2021). Meanwhile, the well-educated had a broad vision to observe problems, whose social resources helped to relieve psychological pressure (Zhang and Bi, 2014). Vocational training also played a great role in eliminating mental poverty (Zhu et al., 2018). It can be seen that strong human capital helped women to establish a good psychological defense and release the psychological pressure caused by career interruption. Thus, the negative correlation between female career interruption and psychological integration was reduced. Moreover, the improvement of female human capital was helpful to overcome the gaps in tradition and institutional constraints (Dustmann, 1996), improving their participation in social security (Gulker and Monteith, 2013). Therefore, women with strong human capital will continue to actively participate in their rights and obligations after career interruption, which ensures institutional integration. However, the loss of female career skills due to career interruption makes it more difficult to meet the escalating skill requirements of new jobs. Therefore, the negative correlation between female career interruption and social integration is more severe for those with limited human capital. Based on the foregoing analysis, we propose the following hypothesis:

**Hypothesis 2:** Human capital positively moderates the relationship between female career interruption and social integration.

### Secondary moderating role of new media use

With the application of digital technology, new media was born. However, the concept of new media lacked a unified definition. For example, United Nations Education Scientific and Cultural Organization (UNESCO) defined new media as the medium for information dissemination based on digital technology and using networks as a carrier. Woo et al. (2014) believed that new media was digital media that can store, transmit, and exchange information through digital binary codes. Sanina (2014) defined new media as an Internet community connecting different forms of mobile communication and electronics. Peng (2016) believed that new media referred to online media, mobile media, and mobile Internet, formed by the fusion of both, as well as other digital media forms with interactivity. The definition of new media in our study is referred from Peng (2016).

Research on new media use can be divided into the following three categories: first, to investigate whether new media were used; second, to measure new media use by some empirical indicators such as frequency and duration (Pinkleton and Austin, 2002); third, scholars turned the perspective on the behavioral characteristics of new media use, such as goals and motivations (Liu et al., 2011; Sundar and Limperos, 2013). Based on the interactivity of new media, the motivation and preference of individuals in the use of new media can also have a differential impact on users. Therefore, this study classified new media use into two dimensions: learning-oriented new media use and amusing-oriented new media use.

Learning-oriented new media use can enhance the weakening effect of human capital on the negative relationship between female career interruption and social integration. When female workers withdrew from the workplace, the effect of human capital appreciation brought by stable work gradually declined (Anderson et al., 2003). However, the skills required by organizations for their employees are constantly improving in the digital economy. Therefore, Lifelong learning was critical (Van Laar et al., 2017). Online learning became an efficient way to improve female employees’ comprehensive quality because of its flexibility (Simonson et al., 2019). Ding and Yuan (2019) also pointed out that compared with women who never surfed the Internet, women who used the Internet had a 63.2% higher probability of learning activities. According to Reinforcement Theory, individuals were more receptive to new things if they continually acquired external dynamic information through online learning to maintain an active mind (Li, 2016). The rational use of new media helped individuals reduce search costs and improve job matching efficiency through online job hunting (Kuhn and Mansour, 2014; Vallas and Schor, 2020). All of these improved the competence of female workers in the labor market, and further enhanced the weakening effect of female human capital on the negative relationship between career interruption and social integration. Meanwhile, learning-oriented new media use could also promote personal self-confidence and social interactions by enriching self-cognition (Alvidrez et al., 2015; Katrin et al., 2019), which shrunk social distance (Lissitsa, 2016). Therefore, a strong learning-oriented new media use can compensate for the hindered social integration after female career interruption due to insufficient human capital. That is, female career interrupters with strong human capital are better able to resist the negative impact of female career interruption when they use new media to learn. Simultaneously, those with limited human capital can supplement new resources and enhance their competence through online learning. Based on the foregoing analysis, we propose the following hypothesis:

**Hypothesis 3:** Learning-oriented new media use positively moderates the moderating effect of human capital on the relationship between female career interruption and social integration.

Amusing-oriented new media use will further weaken the weakening effect of human capital on the negative relationship between female career interruption and social integration. Though the entertainment functions of new media, represented by social chats and communications, played a crucial role in enhancing social connectivity (Kraut et al., 1998), while the functions for leisure, represented by online consumption and Internet games, helped relieve fast-paced life and relax tension (Chan, 2011). However, leisure and entertainment activities represented by new media were often given negative signals such as “be at an idle end” and “not doing honest work” (Rooth, 2011). Employment could promote social integration (Reyes, 2001). However, Mao and Zeng (2017) found that female preference for social and entertainment activities by new media in their leisure time did not significantly promote female overall employment. Moreover, studies proved that the cost of replacing social activities in real life with virtual online socialization is the reduction in time and energy invested in reality (Patton et al., 2016). It was very easy to cause internet addiction and bring a series of negative consequences, including loneliness, depression, anxiety, and social maladjustment (Li et al., 2019). For the female career interrupters, their leisure time may increase when they withdraw from the workplace. If they use new media mainly for entertainment and neglect knowledge supplements during career interruption, it will accelerate the loss of human capital. The more serious consequence is that excessive addiction to the Internet leads to a lack of social communication ability, and even a complete separation from society. This has further exacerbated the gap with mainstream groups caused by female career interruption. Based on the foregoing analysis, we propose the following hypotheses.

**Hypothesis 4:** Amusing-oriented new media use negatively moderates the moderating effect of human capital on the relationship between career interruption and social integration.

## Methodology

### Data collection

This study used the Hainan database of the Fourth Chinese Women’s Social Status Survey in 2020, which is the largest and latest database that can reflect the survival and development of Chinese women. The survey was jointly conducted by the China Women’s Federation and the National Bureau of Statistics. It covered citizens of both sexes of Chinese nationality aged 18–64. In addition to the basic information of the respondents, the database also included aspects on health, education, lifestyle, economy, social security, gender attitude, legal rights, and interest awareness. The data collection process strictly followed the principle of probability sampling to carry out simple random sampling and stratified sampling with equal probability. On this basis, the three-stage sampling method stratified by regional development level was combined with the scale proportional probability sampling (PPS) method to ensure that each sample was representative.

There are 1,800 samples in the Hainan database of the Fourth Chinese Women’s Social Status Survey. Among the samples, 47.3% were female and 52.7% were male. The samples living in urban areas accounted for 60.2%, and the samples living in rural areas accounted for 39.8%.

According to the purpose of this study, we removed all the male samples and invalid samples of choosing “Don’t remember” and “Unable to answer” from the data. According to the Labor Law of China, the legal retirement age for female workers is 50. Therefore, we have eliminated female workers over the age of 50. Thus, a total of 381 valid female samples were finally obtained.

### Measurement tools

#### Social integration

The dependent variable in this study is social integration. When selecting social integration indicators, this study considered the special social problems faced by career interrupters. Drawing on the research basis of social integration by domestic and foreign scholars, 10 question items from the database of the Chinese Women’s Social Status Survey in 2020 were selected as indicators to measure social integration (Table 1). To adapt to social life, one must have sufficient economic income, a certain social status, and a stable occupation (Tian, 1995; Brell et al., 2020). Thus, three economic indicators were selected: income level, occupational class, and occupational stability. Given that many scholars emphasized the importance of social networks and social relationships in the community for integration (Mai and Wang, 2022), social group participation was selected as an indicator to measure cultural integration. Based on the Chinese long-standing advocacy of gender equality, women were encouraged to participate in politics and show their responsibility. Therefore, the willingness to become a community member was also selected to measure cultural integration. Psychological integration is also crucial (Schick et al., 2016). Therefore, three psychological indicators were selected to measure psychological integration: confidence in life, ability to deal with life issues, and interaction with people. The most prominent issue in the institutional aspect is the public service system, with social security and medical care as the key ones. The choice of social pension insurance can significantly affect social integration (Zhang et al., 2015), and the public service system represented by hospitals played an important role in social integration (Liu et al., 2021). Thus, the purchase of social pension insurance and social health insurance were selected to measure institutional integration.

**TABLE 1:** Descriptive statistics of social integration indicators.

Dimension		Indicator measurement	Range	Mean	SD
Economic integration	Y_1_	Income level	0∼3	1.69	1.38
	Y_2_	Occupational class	0∼3	2.10	0.63
	Y_3_	Occupational stability	0∼3	0.91	0.97
Cultural integration	Y_4_	Social group participation	0∼5	0.49	0.88
	Y_5_	Willingness to become a community member	0∼1	0.29	0.45
Psychological integration	Y_6_	Confidence in life	1∼4	3.41	0.62
	Y_7_	Ability to deal with life issues	1∼4	3.20	0.62
	Y_8_	Interaction with people	1∼4	3.51	0.55
Institutional integration	Y_9_	Purchase of social pension insurance	0∼1	0.73	0.45
	Y_10_	Purchase of social medical insurance	0∼1	0.97	0.18

Social integration was measured using factor analysis. The weights of the variance contribution ratios corresponding to each common factor were used, and the composite scores were calculated, which were finally linearly transformed using the min-max standardization method and converted to values in [0, 100]. A higher score indicate deeper social integration. The descriptive statistics of social integration and scores of each dimension are represented in Table 2.

**TABLE 2:** Social integration and scores of each dimension.

Dimension	Mean	SD	*N*	Minimum	Maximum
Economic integration	53.99	21.63	381	0	100
Cultural integration	26.50	16.51	381	1.98	100
Psychological integration	62.93	18.72	381	0	97.21
Institutional integration	79.78	17.09	381	0	100
Social integration	58.59	13.22	381	-0.12	99.99

#### Female career interruption

The independent variable in this study is female career interruption. It was measured by the duration of female career interruption, which corresponded to the original question items in the questionnaire to investigate the specifics of the respondent’s career interruption.

#### Human capital

The moderating variable in this study is human capital. It was measured by education (1 = elementary school and below, 2 = junior high school, 3 = high school/junior college, 4 = college and above), self-rated health status (1 = very poor, 2 = poor, 3 = fair, 4 = good, 5 = very good), and participation in training in the last 3 years (1 = 0 times, 2 = once, 3 = two to five times, 4 = six times and above) in the questionnaire. Specifically, human capital was obtained by summing and averaging the scores of these three indicators. A higher score indicates stronger human capital.

#### New media use

The secondary moderating variable in this study is new media use. This corresponds to the original question in the questionnaire that investigates respondents’ online behavior. The options included learning about news, working/business, studying online, investing/financial management, online consumption, chatting, entertainment, and games. Respondents were limited to two of these options. Considering the online behavior represented by each option, new media use was classified as learning-oriented and amusing-oriented.

Learning-oriented new media use. The options for learning about news, working/business, studying online, and investing/financial management were incorporated into learning-oriented new media use. One of them was assigned a score of 1, and a variable of (0, 1, 2) was derived by adding equal weights to measure the new media use-learning orientation.

Amusing-oriented new media use. The options of online consumption, chatting, entertainment, and games were incorporated into the amusing-oriented new media use. The specific quantification of this variable is consistent with learning-oriented new media use.

#### Control variables

To control the influence of individual characteristics, according to previous studies, age, urban and rural attributes, and marriage were used as control variables. Age was a continuous variable, and the classified variables of urban and rural attributes and marriage are transformed into dummy variables.

### Descriptive statistics

Data descriptions of the variables are listed in Table 3.

**TABLE 3:** Statistical description of variables.

Variable	Code	Range	Mean	SD
**Dependent variable**				
Social integration	SI	−0.12–99.99	58.59	13.22
**Independent variable**				
Female career interruption	CI	6–204	13.77	30.21
**Moderating variable**				
Human capital	HC	1–3.75	1.84	0.58
Learning-oriented new media use	learning	0–2	0.70	0.72
Amusing-oriented new media use	amusing	0–2	1.26	0.73
**Control variable**				
Age	AGE	18–50	34.40	7.96
Urban and rural attributes	URBAN	Dummy variable (urban = 1)	1.24	/
Marriage	MARRIAGE	Dummy variable (married = 1)	1.78	/

### Regression model

To test hypothesis 1, the following formula was developed in this study:


(1) $$S\,I_{i}=\alpha +\beta \,C\,I_{i}+\theta \,X_{i}+\varepsilon_{i}$$

*SI*_*i*_ is the dependent variable, indicating the social integration score. *CI*_*i*_ is the independent variable, indicating female career interruption. *X_i* represents the control variables.

To test Hypothesis 2, this study introduced an interaction term between female career interruption and human capital using the following formula:


(2) $$S\,I_{i}=\alpha +\beta \,C\,I_{i}+\rho \,H\,C_{i}+\eta \,C\,I_{i}\times H\,C_{i}+\theta \,X_{i}+\varepsilon_{i}$$

The meanings of *SI*_*i*_,*CI*_*i*_,*X*_*i*_ in Equation 2 are the same as those in Equation 1. *HC*_*i*_ is the moderating variable and denotes human capital. *CI*_*i*_×*HC*_*i*_ denotes the interaction term between career interruption and human capital. If the coefficient of *CI*_*i*_×*HC*_*i*_ is significantly positive, Hypothesis 2 is valid. Otherwise, Hypothesis 2 cannot be verified.

To test Hypotheses 3 and 4, this study introduced a three-way interactive term for career interruption, human capital, and new media use.


(3) $$S\,I_{\text{i}}=\alpha +\beta \,C\,I_{i}+\rho \,H\,C_{i}+\omega \,l\,e\,a\,r\,n\,i\,n\,g_{i}+\eta \,C\,I_{i}\times H\,C_{i}+\gamma \,C\,I_{i}$$

$$\times l\,e\,a\,r\,n\,i\,n\,g_{i}+\chi \,H\,C_{i}\times l\,e\,a\,r\,n\,i\,n\,g_{i}+\xi \,C\,I_{i}\times H\,C_{i}\times l\,e\,a\,r\,n\,i\,n\,g_{i}$$

$$+\theta \,X_{i}+\varepsilon_{i}$$

(4) $$S\,I_{i}=\alpha +\beta \,C\,I_{i}+\rho \,H\,C_{i}+\omega \,a\,m\,u\,s\,i\,n\,g_{i}+\eta \,C\,I_{i}\times H\,C_{i}+\gamma \,C\,I_{i}$$

$$\times a\,m\,u\,s\,i\,n\,g_{i}+\chi \,H\,C_{i}\times a\,m\,u\,s\,i\,n\,g_{i}+\xi \,C\,I_{i}\times H\,C_{i}$$

$$\times a\,m\,u\,s\,i\,n\,g_{i}+\theta \,X_{i}+\varepsilon_{i}$$

*learning*_*i*_ in Equation 3 was the secondary moderating variable, indicating the learning-oriented new media use. *CI*_*i*_×*HC*_*i*_×*learning*_*i*_ represents the three-way interactive term of female career interruption, human capital, and learning-oriented new media use. If the coefficient of this term is significantly positive, Hypothesis 3 is valid.

Similarly, *amusing*_*i*_ in Equation 4 is the secondary moderating variable indicating amusing-oriented new media use. *CI*_*i*_×*HC*_*i*_×*amusing*_*i*_ represents the three-way interactive term of female career interruption, human capital, and amusing-orientation new media use. If the coefficient of this term is significantly positive, Hypothesis 4 is valid.

To verify the hypothesis, OLS was first used to test the hypotheses. However, OLS can only get the influence of conditional expectations among variables, the estimation results are vulnerable to extreme values. Therefore, to improve the accuracy of model estimation and avoid the influence of extreme values on results, quantile regression was applied in this study. Koenker and Bassett (1978) put forward the quantile regression method. Quantile regression assumed that the quantile of the conditional distribution of the dependent variable is a linear function of the independent variable. The quantile regression model was constructed to reveal the influence of independent variables on the distribution of the dependent variable.

## Data analysis

### Female career interruption and social integration

Equation 1 was the first to explore the relationship between female career interruption and social integration. Then quantile regression was used to further investigate the heterogeneous correlation between female career interruption and social integration. Results were presented in Table 4.

**TABLE 4:** Female career interruption and social integration.

Variable	(1)			
	OLS	q25	q50	q75
CI	−0.037* (−1.965)*^a^*	−0.068* (−1.997)	−0.009 (−0.264)	−0.048^+^ (−1.711)
AGE	−0.023 (−0.245)	0.039 (0.296)	−0.065 (−0.511)	−0.026 (−0.199)
MARRIAGE	0.801 (0.510)	1.552 (0.657)	−0.954 (−0.445)	−1.453 (−0.672)
URBAN	−7.675*** (−4.817)	−7.157** (−3.218)	−7.741** (−3.562)	−9.767*** (−4.455)
*R* ^2^	0.064	0.032	0.042	0.054
*F*	6.445***	-	-	-

^+^p < 0.1, *p < 0.05, **p < 0.01, ***p < 0.001.

^a^The figures in brackets of tables are t-values. The same as follows.

The OLS regression results in Table 4 shows that there is a significant negative correlation between female career interruption and social integration (β = −0.037, ρ < 0.05). For every 1 SD increase in female career interruption, social integration decreases by 0.037 points. Hypothesis 1 is verified. However, OLS cannot reflect the regular distribution of the influence of independent variables on the growth of the dependent variable. Therefore, quantile regression was applied. Table 4 presents the regression results of social integration at 25, 50, and 75% quantile. From the quantile regression results in Table 4, it can be seen that there were significant differences in the link between female career interruption and social integration at different quantiles. At the 25 and 75% quantiles, the negative correlation between female career interruption and social integration is significant (β_*q25*_ = −0.067, ρ < 0.05; β_*q75*_ = −0.048, ρ < 0.1). However, the negative correlation between female career interruption and social integration is not significant at the 50% quantile (β_*q50*_ = −0.009, n. s.). It shows that female career interruption is more likely to lead to a social disintegration when the social integration is low or high than when the social integration is moderate.

### Analysis of the moderating effects

#### Moderating role of human capital

Equation 2 was used to explore the moderating effect of human capital on the relationship between female career interruption and social integration. Then quantile regression was used to further investigate the heterogeneity. Results were presented in Table 5.

**TABLE 5:** Analysis of the moderating impact of human capital.

Variable	(2)				
	OLS		q25	q50	q75
CI	−0.009 (−0.431)	−0.028 (−0.987)	−0.148* (−3.149)	−0.012 (−0.307)	−0.011 (−0.250)
HC	9.064*** (7.811)	8.851*** (7.503)	7.476*** (4.343)	9.024*** (6.260)	11.399*** (6.806)
CI × HC		−0.045 (−0.998)	−0.117 (−1.551)	−0.025 (−0.662)	−0.097 (−1.320)
AGE	0.093 (1.048)	0.084 (0.937)	0.111 (0.872)	0.115 (1.025)	0.165 (1.330)
MARRIAGE	1.329 (0.911)	1.570 (1.062)	3.947 (1.840)	3.240 (1.804)	−1.272* (−0.609)
URBAN	−3.372* (−2.136)	−3.316* (−2.099)	−2.901 (−1.273)	−4.827* (−2.528)	−5.137* (2.316)
*R* ^2^	0.195	0.197	0.110	0.122	0.138
Δ*R*^2^	0.195	0.002	-	-	-
*F*	18.184***	15.319***	-	-	-

*p < 0.05, ***p < 0.001.

The OLS regression results in Table 5 show that the link between human capital and social integration is significantly positive (β = 8.851, ρ < 0.001), for every 1 SD increase in human capital, social integration increased by 8.851 points. However, the interaction between female career interruption and human capital is not significant (β = −0.045, n. s.). Hypothesis 2 is not verified. Table 5 also presents the regression results of social integration at 25, 50, and 75% quantile. However, the quantile regression in Table 5 indicates that the interaction between female career interruption and human capital has no significant impact on social integration at different quantiles (β_*q25*_ = −0.117, n. s.; β_*q50*_ = −0.025, n. s.; β_*q75*_ = −0.097, n. s.). One possible explanation is that although women with strong human capital were well-integrated into society, due to the lack of necessary external support (e.g., good study habits, and information channels), strong human capital cannot offset the negative relationship between career interruption and social integration.

#### Secondary moderating role of learning-orientated new media use

Table 6 presents OLS and quantile regression results of the secondary moderating effect of learning-oriented new media use. The OLS regression results in Table 6 show that the coefficient of learning-oriented new media use was significantly positive (β = 1.643, ρ < 0.05), for every 1 SD increase in learning-oriented new media use, social integration increases by 1.643 points. And the three-way interactive term of female career interruption, human capital and learning-oriented new media use was also significantly positive (β = 0.247, ρ < 0.01). Learning-oriented new media use can further enhance the weakening effect of human capital on the negative relationship between female career interruption and social integration. Hypothesis 3 is verified. Table 6 also shows the regression results of social integration at the 25, 50, and 75% quantile. The quantile regression indicates that on the 25 and 50% quantiles of social integration, the three-way interactive term of female career interruption, human capital and learning-oriented new media use had a significantly positive impact on social integration (β_*q25*_ = 0.329, ρ < 0.01; β_*q50*_ = 0.243, ρ < 0.05). However, with the improvement of social integration, the absolute value of the three-way interactive term coefficient gradually decreased. Finally, the impact of the three-way interactive term on social integration is no longer significant at 75% quantile (β_*q75*_ = 0.155, n. s.). For female career interrupters with lower social integration, learning-oriented new media use can better enhance the role of human capital. For female career interrupters with higher social integration, the improvement effect brought by learning-oriented new media use was not obvious, which showed that the use of new media for learning can play a limited role in female career interrupters with high a degree of social integration.

**TABLE 6:** Analysis of the secondary moderating impact of learning-oriented new media use.

Variable	(3)					
		OLS		q25	q50	q75
CI	−0.008 (−0.387)	−0.005 (−0.168)	−0.005 (−0.182)	−0.011 (−0.227)	−0.008 (−0.215)	−0.021 (−0.416)
HC	8.732*** (7.364)	8.509*** (7.031)	9.160*** (7.504)	10.837*** (6.633)	8.864*** (5.976)	8.345*** (4.673)
Learning	1.174^+^ (1.325)	1.102* (1.435)	1.643* (1.817)	3.985** (3.595)	0.568 (0.528)	0.588 (0.454)
CI × HC		−0.037 (−0.812)	−0.028 (−0.614)	−0.041 (−0.577)	−0.047 (−0.731)	−0.158* (−2.049)
CI × learning		0.074^+^ (1.888)	0.159** (3.242)	0.125^+^ (1.852)	0.176** (2.873)	0.213** (2.886)
HC × learning		1.683 (1.025)	3.350* (2.101)	3.608^+^ (1.708)	4.540* (2.873)	5.031* (2.178)
CI × HC × learning			0.247** (2.829)	0.329** (2.755)	0.243* (2.247)	0.155 (1.192)
AGE	0.084 (0.940)	0.042 (0.455)	0.054 (0.597)	0.209^+^ (1.762)	0.068 (0.635)	0.006 (0.045)
MARRIAGE	1.055 (0.717)	1.425 (0.954)	1.595 (1.087)	2.907 (1.486)	4.011* (2.259)	0.359 (0.169)
URBAN	−3.584* (−2.261)	−3.650* (−2.304)	−3.245* (−2.059)	−2.788 (−1.347)	−2.658 (−1.414)	−4.237 (−1.872)
*R* ^2^	0.199	0.209	0.226	0.156	0.155	0.157
Δ*R*^2^	0.199	0.010	0.017	-	-	-
*F*	15.476***	10.918***	10.812***	-	-	-

^+^p < 0.1, *p < 0.05, **p < 0.01, ***p < 0.001.

#### Secondary moderating role of amusing-orientated new media use

Table 7 showed OLS and quantile regression results of the secondary moderating effect of amusing-oriented new media use. The OLS regression results in Table 7 showed that the coefficient of amusing-oriented new media use was significantly negative (β = −1.657, ρ < 0.1), for every 1 SD increase in amusing-oriented new media use, social integration decreased by 1.657 points. And the three-way interactive term of female career interruption, human capital and amusing-oriented new media use was also significantly negative (β = −0.245, ρ < 0.05). Amusing-oriented new media use can further weaken the weakening effect of human capital on the negative relationship between female career interruption and social integration. Hypothesis 4 is verified. Table 7 also presents the regression results of social integration at the 25, 50, and 75% quantile. The quantile regression showed that on the 25 and 50% quantiles of social integration, the three-way interactive term of female career interruption, human capital and amusing-oriented new media use had a significantly negative impact on social integration (β_*q25*_ = −0.336, ρ < 0.05; β_*q50*_ = −0.252, ρ < 0.05). However, with the improvement of social integration, the absolute value of the three-way interactive term coefficient gradually decreased. Finally, the impact of the three-way interactive term on social integration is no longer significant at 75% quantile (β_*q75*_ = −0.191, n. s.). For female career interrupters with lower social integration, amusing-oriented new media use can further inhibit the role of human capital. For female career interrupters with higher social integration, the inhibition brought by amusing-oriented new media use was not obvious, which showed that the use of new media for amusement can play a limited role in female career interrupters with high a degree of social integration.

**TABLE 7:** Analysis of the secondary moderating impact of amusing-oriented new media use.

Variable	(4)					
		OLS		q25	q50	q75
CI	−0.009 (−0.407)	−0.019 (−0.672)	−0.032 (−1.094)	−0.010 (−0.204)	−0.060^+^ (−1.444)	−0.023 (−0.466)
HC	8.819*** (7.499)	8.506*** (7.033)	8.828*** (7.290)	10.938*** (6.129)	9.229*** (6.139)	8.138*** (4.633)
amusing	−1.175^+^ (−1.245)	−1.075 (−1.239)	−1.657^+^ (−1.842)	−3.890*** (−2.963)	−1.470 (−1.330)	−0.470 (−0.364)
CI × HC		−0.046 (−1.004)	−0.059 (−1.299)	0.047 (0.615)	−0.091 (−1.409)	−0.149* (−1.970)
CI × amusing		−0.046^+^ (−1.124)	−0.139* (−2.417)	−0.119 (−1.359)	−0.189* (−2.558)	−0.233*** (−2.698)
HC × amusing		−1.086 (−0.743)	−2.925^+^ (−1.759)	−3.499 (−1.440)	−4.001* (−1.995)	−5.988* (−2.504)
CI × HC × amusing			−0.245* (−2.274)	−0.336* (−2.076)	−0.252* (−2.582)	−0.191 (−1.199)
AGE	0.082 (0.917)	0.047 (0.513)	0.041 (0.448)	0.216 (1.626)	0.065 (0.585)	0.085 (0.651)
MARRIAGE	1.097 (0.746)	1.551 (1.030)	1.802 (1.200)	3.234 (1.470)	3.025 (1.632)	−0.168 (−0.078)
URBAN	−3.508* (−2.218)	−3.612* (−2.272)	−3.319* (−2.090)	−3.033 (−1.312)	−3.160 (−1.623)	−4.007^+^ (−1.761)
*R* ^2^	0.199	0.204	0.215	0.143	0.143	0.157
Δ*R*^2^	0.199	0.005	0.011	-	-	-
*F*	15.433***	10.592***	10.217***	-	-	-

^+^p < 0.1, *p < 0.05, ***p < 0.001.

### Heterogeneity analysis of different social integration dimensions

This study divided social integration into four dimensions: economic integration, cultural integration, psychological integration, and institutional integration. Furthermore, female career interrupters may have some differences in their economic, cultural, psychological, and institutional integration. Therefore, based on the above regression model, the dependent variable social integration was replaced by each dimension of social inclusion (economic integration, cultural integration, psychological integration, and institutional integration) to further test the relationship between female career interruption and different dimensions of social inclusion. The results were shown in Table 8.

**TABLE 8:** Heterogeneity analysis of different social integration dimensions.

Variable	Economic integration				Cultural integration				Psychological integration				Institutional integration			
CI	−0.078* (−2.288)	−0.081^+^ (−1.858)	−0.039 (−0.839)	−0.035 (−1.035)	−0.030 (−1.091)	−0.002 (−0.063)	−0.008 (−1.121)	−0.011 (−1.051)	−0.033 (−1.029)	−0.021 (−0.499)	−0.010 (−0.196)	−0.041 (−0.713)	0.043 (1.439)	0.047 (1.252)	0.036 (1.245)	0.056 (1.478)
HC		12.359*** (6.784)	11.935*** (6.292)	9.355*** (5.952)		4.559** (2.941)	3.597* (2.226)	3.870* (2.363)		7.872*** (4.406)	8.671*** (4.721)	8.698*** (4.876)		1.259 (1.857)	0.985 (0.957)	0.322 (0.345)
learning			0.968^+^ (1.687)				0.738 (0.616)				1.737^+^ (1.276)				1.392 (1.708)	
amusing				−1.297 (−1.101)				0.833 (0.965)				−1.590 (−1.088)				−2.020^+^ (−1.826)
CI × HC		0.090^+^ (1.622)	0.082 (1.377)	0.098 (1.447)		0.033 (0.567)	0.042 (0.702)	0.053 (0.919)		0.031 (0.452)	0.040 (0.540)	0.044 (0.682)		0.036 (1.264)	0.021 (1.182)	0.004 (0.057)
CI × learning			0.144^+^ (1.881)				−0.074 (−1.143)				0.222** (3.005)				0.078 (1.598)	
CI × amusing				−0.123 (−1.011)				0.148^+^ (2.255)				−0.221* (−3.011)				−0.098 (−1.325)
HC × learning			3.763^+^ (1.516)				3.575^+^ (1.681)				6.542* (2.311)				−3.870* (−2.092)	
HC × amusing				−3.624^+^ (−1.921)				−1.297 (−0.753)				−5.584* (−2.426)				5.678* (2.731)
CI × HC × learning			0.004 (0.035)				−0.160 (−1.383)				0.459** (3.497)				0.079 (0.783)	
CI × HC × amusing				−0.005 (−0.066)				0.220^+^ (1.621)				−0.425** (−3.013)				−0.132 (−0.871)
AGE	−0.295* (−3.554)	−0.152 (−1.105)	−0.232 (−1.638)	−0.252** (−2.077)	−0.470*** (−4.025)	−0.406** (−3.476)	−0.472*** (−3.922)	−0.390** (−2.567)	0.521*** (3.858)	0.616*** (4.5897)	0.592*** (4.322)	0.489*** (3.985)	−0.094 (−0.241)	−0.011 (−0.290)	−0.048 (0.578)	−0.054 (0.867)
MARRIAGE	−6.612** (−2.755)	−5.319* (−2.328)	−5.303* (−2.301)	−5.713* (−2.403)	10.242*** (5.302)	10.318*** (5.309)	10.243*** (5.229)	9.988*** (5.098)	−1.719 (−0.764)	−1.090 (−0.489)	−0.849 (−0.381)	−1.507 (−0.837)	0.823 (0.434)	0.733 (0.397)	0.696 (0.353)	0.719 (0.367)
URBAN	−18.170*** (−7.457)	−11.948*** (−4.897)	−12.403*** (−5.054)	−12.044*** (−5.056)	−6.129*** (−3.126)	−4.082^+^ (−1.967)	−4.457* (−2.137)	−3.418* (−1.919)	1.195 (0.523)	5.018* (2.109)	5.481* (2.312)	5.243* (2.214)	−0.123 (−0.066)	−0.230 (−0.186)	−0.536 (−0.334)	−0.385 (−0.276)
*R* ^2^	0.182	0.284	0.299	0.318	0.090	0.111	0.130	0.093	0.040	0.092	0.126	0.114	0.009	0.011	0.047	0.052

^+^p < 0.1, *p < 0.05, **p < 0.01, ***p < 0.001.

#### Female career interruption and economic integration

It can be seen from Table 8 that there is a significant negative correlation between female career interruption and economic integration (β = −0.078, ρ < 0.05), for every 1 SD increase in female career interruption, economic integration decreased by 0.078 points. When human capital and the interaction between female career interruption and human capital were incorporated, the coefficient of interaction between female career interruption and human capital was significantly positive (β = 0.090, ρ < 0.1). The moderating role of human capital in the relationship between female career interruption and economic integration was supported. To further test the secondary moderating effect of new media use, the three-way interactive term of female career interruption, human capital, and learning-oriented new media use was not significant (β = 0.004, n. s.), and the three-way interactive term of female career interruption, human capital, and amusing-oriented new media use was not significant too (β = −0.005, n. s.). The secondary moderating effect of new media use was not supported. It showed that female career interruption and human capital were the important factors affecting female economic integration. However, new media use played a limited role as the secondary moderating variable in the relationship between female career interruption and economic integration.

#### Female career interruption and cultural integration

It can be seen from Table 8 that the relationship between female career interruption and cultural integration was not significant due to the limited role of career interruption in inhibiting female cultural integration (β = −0.030, n. s.). When human capital and the interaction between female career interruption and human capital were incorporated, the coefficient of interaction between female career interruption and human capital was not significant (β = 0.033, n. s.). The moderating role of human capital in the relationship between female career interruption and cultural integration was not supported, which indicated that human capital cannot weaken the negative link between female career interruption and cultural integration. To further test the secondary moderating effect of new media use, the three-way interactive term of female career interruption, human capital, and learning-oriented new media use was not significant (β = −0.160, n. s.), but the three-way interactive term of female career interruption, human capital, and amusing-oriented new media use was significantly positive (β = 0.220, ρ < 0.1). The possible reason was that the social function covered by learning-oriented helped women to accumulate social capital (Kuhn and Mansour, 2014) and enhance social connection (Kraut et al., 1998), thus promoting female cultural integration.

#### Female career interruption and psychological integration

Table 8 showed that the link between female career interruption and psychological integration was not significant due to the limited role of career interruption in inhibiting female psychological integration (β = −0.033, n. s.). When human capital and the interaction between female career interruption and human capital were incorporated, the coefficient of interaction between female career interruption and human capital was not significant (β = 0.031, n. s.). The moderating role of human capital in the relationship between female career interruption and psychological integration was not supported, which indicated that human capital cannot weaken the negative link between female career interruption and psychological integration. To further test the secondary moderating effect of new media use, the three-way interactive term of female career interruption, human capital, and learning-oriented new media use was significantly positive (β = 0.459, ρ < 0.01). And the three-way interactive term of female career interruption, human capital, and amusing-oriented new media use was significantly negative (β = −0.425, ρ < 0.01). The secondary moderating effect of new media use was supported.

#### Female career interruption and institutional integration

Table 8 showed that the link between female career interruption and institutional integration was not significant (β = 0.043, n. s.). The interaction between female career interruption and human capital was not significant (β = 0.036, n. s.). Moreover, the three-way interactive term of female career interruption, human capital, and learning-oriented new media use was not significant (β = 0.079, n. s.), and the three-way interactive term of female career interruption, human capital, and amusing-oriented new media use was not significant (β = −0.132, n. s.). The possible explanation was that the social institution was a compulsory tool implemented by the country for citizens, and the purpose was to ensure that all citizens can participate in it fairly and without exclusion. Therefore, regardless of the level of human capital and the new media use, the institutional integration cannot be significantly affected by individual factors under the country’s compulsory requirements.

## Research results and discussion

### Research results

This study explored the relationship between female career interruption and social integration, and then discussed the moderating role of human capital and new media use. Through the foregoing data analysis, the relationship between the variables was verified, and the following hypotheses were confirmed:

1. The relationship between female career interruption and social integration is significantly negative. Through quantile regression, it was found that female career interruption is more likely to lead to a social disintegration when the social integration is low or high than when the social integration is moderate. From the perspective of different dimensions of social integration, the link between female career interruption and economic integration is significant negative, and the correlation in cultural, psychological, and institutional integration was not significant. Specifically, the link between female career interruption and social integration was mainly reflected in economic integration. This is consistent with previous research findings. Female career interruption led to the decline of female workers’ wages (Viitanen, 2014; Ali et al., 2020) and restricted career development (Engelhardt et al., 2004; Mandel and Semyonov, 2006), which can harm women’s economic integration. However, the link in cultural, psychological, and institutional integration was not significant. It may be that after female career interrupted, due to the increase in leisure time, they can invest more time participating in the interaction with relatives, friends, and neighbors. To a certain extent, this interaction made female cultural, psychological, and institutional integration less vulnerable to career interruption.
2. Human capital plays a moderating role in the relationship between female career interruption and economic integration, which can weaken the correlation between female career interruption and economic integration. Previous studies confirmed that human capital promotes individual social integration by increasing income. For example, Huang et al. (2018) found that women can effectively improve their income after higher education. Enhancing knowledge and skills can increase return on revenue and promote integration into society (Long, 1980; Borjas, 1982). In the past, these studies focused on the positive role of human capital in working groups. Our study examines and verifies the unique positive moderating role of human capital in the relationship between female career interruption and economic integration from the perspective of female career interrupters. In addition, the study found that human capital did not play a significant role as a moderating variable in the relationship between female career interruption and social integration, indicating that the role of human capital in offsetting the negative relationship between female career interruption and social integration is limited.
3. However, the impact of human capital on the link between female career interruption and social integration was dependent on new media use. Specifically, when women with strong learning-oriented new media use encountered human capital, the relationship between female career interruption and social integration was minimal. In contrast, this relationship was enhanced when women with strong amusing-oriented new media use were confronted with human capital. The results of quantile regression showed that the secondary moderating effects of learning-oriented new media use and amusing-oriented new media use gradually were weakened with the rise of the quantile of female social integration. From different dimensions of social integration, learning-oriented new media use enhanced the weakening effect of human capital on the negative correlation between female career interruption and psychological integration, while amusing-oriented new media use weakened the weakening effect of human capital on the negative correlation between female career interruption and psychological integration. However, amusing-oriented new media use can enhance the weakening effect of human capital on the negative correlation between female career interruption and cultural integration. Previous studies had confirmed that the social functions covered by amusing-oriented new media use helped to increase social connectivity (Kraut et al., 1998), thus promoting female cultural integration.

### Theoretical contributions

The theoretical contributions of this study are as follows.

First, it enriches the theoretical research of social integration. Departed studies on social integration mainly focused on migrants (Snel et al., 2006), migrant workers, and other floating populations (Yang, 2009). Less attention has been paid to female career interrupters. In addition, most existing studies on the factors affecting social integration were limited to human capital, social capital, and institutional structure (Li and Tian, 2012), while the research on the relationship between career interruption and social integration was very limited. This study analyzes social integration from the perspective of female career interruption. Based on social integration theory and human capital theory, we test the relationship between female career interruption and social integration through theoretical derivation and empirical analysis. The results reveal that the relationship between female career interruption and social integration was negative. And for women with different degrees of social integration, the relationship between female career interruption and social integration was different, which enriched the theoretical research of social integration.

Second, it refines the theoretical research on new media use. In previous studies on new media use, the measurement was relatively general. Most studies aimed to investigate the impact of whether to use new media on individuals, lacking further refinement of new media use behavior. This study complements it by dividing specific new media use behaviors into learning-oriented new media use and amusing-oriented new media use according to the interactivity of new media, which brings human capital and new media use into one research framework at the same time. Based on new media use behaviors with different preferences or motives, this study explores the differential impacts of human capital on the relationship between female career interruption and social integration. It refines the theoretical research on new media use and provides more interpretations of the relevant theories of social integration.

### Practical contributions

This study’s findings are beneficial for understanding the underlying mechanisms of human capital and new media use that influence the relationship between female career interruption and social integration. It provides references for women who experienced career interruption to effectively enhance their competitiveness and promote social integration. (1) Considering the negative link between female career interruption and social integration, the government should make efforts to protect women’s rights and interests. First of all, the government should list fertility friendliness as a considerable evaluation of employers’ social responsibility, and encourage employers to formulate measures that are conducive to balancing the relationship between female work and family. It is also necessary to negotiate a flexible work system profitable for the care of infants and young children by law. Improve the laws and regulations on female labor protection, and implement the maternity leave and lactation leave system. Punishment should be applied to the employers for reducing female wages and dismissing female employees due to pregnancy, childbirth, and lactation, to increase the economic compensation for women and the illegal costs of enterprises. Also, it is necessary to further optimize the policy of supporting women’s childbirth to ensure that the childbearing medical expenses and childbearing allowances of maternity insurance for female workers are commensurate with the burden of childcare. In addition, specific anti-employment discrimination laws must be issued, requiring employers not to directly define gender in their recruitment ADs. In case of special circumstances, it must be fully justified to legally guarantee equal employment opportunities and conditions for all female workers. The employment department should also provide re-employment guidance and training for female career interrupters, helping to rebuild their employment skills. (2) Human capital and new media use are both important resources for social integration, and the influence of both is inextricably linked. Reasonably guiding female career interrupters to use new media boosts human capital playing a positive role in helping women better integrate into society. Therefore, during career interruption, on the one hand, female workers should improve professionalism by actively participating in knowledge accumulation according to their human capital; on the other hand, they should supplement timely information and acquire new skills through online learning. The correlation between female career interruption and social integration can be effectively mitigated if they develop good habits for strong learning-oriented new media use. Although, amusing-oriented new media use in a reasonable way can help eliminate the stress and anxiety caused by unfamiliar environments. However, if left uncontrolled, this may result in the rise of socially isolated human beings, which is adverse to social integration.

### Research limitations and future prospects

This study makes theoretical and practical contributions to understanding the relationship between female career interruption and social integration. However, it has some limitations. First, the sample was obtained from the data of the Fourth Chinese Women’s Social Status Survey in 2020 in Hainan Province. Given the differences in job opportunities availability, income level, and female education level of Hainan province, whether the results apply to other countries or regions requires further study. Second, this study used second-hand cross-sectional data, which made it difficult to prove the causal relationship between these variables. Future research can use tracking data to explore deeper relationships among variables through a longitudinal comparison.

## Data availability statement

The original contributions presented in this study are included in the article/supplementary material, further inquiries can be directed to the corresponding author.

## Ethics statement

Ethical review and approval was not required for the study on human participants in accordance with the local legislation and institutional requirements. Written informed consent from the patients/participants OR patients/participants legal guardian/next of kin was not required to participate in this study in accordance with the national legislation and the institutional requirements.

## Author contributions

All authors listed have made a substantial, direct, and intellectual contribution to the work, and approved it for publication.
